# Fatigue, Work Overload, and Sleepiness in a Sample of Spanish Commercial Airline Pilots

**DOI:** 10.3390/bs13040300

**Published:** 2023-04-01

**Authors:** Ana Alaminos-Torres, Jesús Román Martínez-Álvarez, Manuela Martínez-Lorca, Noemí López-Ejeda, María Dolores Marrodán Serrano

**Affiliations:** 1 Physical Anthropology Unit, Department of Biodiversity, Ecology and Evolution, Faculty of Biological Sciences, Complutense University of Madrid, 28040 Madrid, Spain; noemilop@ucm.es; 2 EPINUT Research Group, Faculty of Medicine, Complutense University of Madrid, 28040 Madrid, Spain; 3 Spanish Society of Dietetics and Food Sciences, Pozuelo de Alarcón, 28224 Madrid, Spain; 4 Department of Psychology, Faculty of Health Sciences, University of Castilla-La Mancha, 45600 Talavera de la Reina, Spain

**Keywords:** fatigue, work overload, daytime sleepiness, aviation safety, airline pilots, raw TLX questionnaire, fatigue severity scale, Epworth sleepiness scale questionnaire

## Abstract

Commercial aviation pilots are an occupational group that work in particular conditions, with frequent schedule changes, shift work, unfavorable environmental conditions, etc. These circumstances can lead to fatigue, work overload (WO), and daytime sleepiness, factors that can affect their health and safety. This study aimed to assess the prevalence and the association between these parameters in a sample of Spanish commercial airline pilots. The Raw TLX, Fatigue Severity Scale, and the Epworth Sleepiness Scale questionnaires were administered in a sample of 283 participants. The relationships of the total scores between all the questionnaires were studied by the chi-square test and the risk scores (odds ratio) were calculated. Different models using multiple linear regression were carried out to evaluate the effects of WO, fatigue, and daytime sleepiness, among the total scores, age, and flight hours. Additionally, the internal consistency of each questionnaire was estimated. A total of 28.2% presented WO above the 75th percentile, with mental and temporal demand the dimensions with the greatest weight. A total of 18% of pilots presented fatigue, 15.8% moderate sleepiness, and 3.9% severe sleepiness. We observed an association among WO, fatigue, and daytime sleepiness, important factors related to pilot health and aviation safety.

## 1. Introduction

Fatigue is one of the most critical human factors that impact aviation safety [1]. It has been defined by the International Civil Aviation Organization [2] as “a physiological state of reduced mental or physical performance capability resulting from sleep loss or extended wakefulness, circadian phase, or workload (mental and/or physical activity) that can impair a crewmember’s alertness and ability to safely operate an aircraft or perform safety related duties”. Among commercial airline pilots, fatigue can reduce flight operational performance [3,4], increase the risk for an incident or even an accident [5], and can contribute to cognitive performance during a flight [6]. 

According to the International Organization for Standardization, workload is the set of external conditions and requirements in a work system, which affect the physiological and/or psychological state of a person [7]. Work overload (WO) is one of the most important predictors of burnout, and different approaches to their study have shown common factors such as the qualitative and quantitative demands of the job, the lack of control, reward, or social support, among others [8]. 

Among the factors that may contribute to the development of burnout in aviation pilots are reduced rest periods, aircraft issues, pressure to meet on time performance goals, or adverse weather conditions [9]. Additionally, the relationship between burnout and psychosocial characteristics was studied by Demerouti et al. [10], who showed that pilot burnout was also related to decreased happiness or well-being, which influences the pilots’ life and with worse simulator training performance. Che et al. [11] analyzed how mental work overload was a key factor in risk analysis and accident prevention. Workload can reduce alertness and vigilance, increasing the likelihood of accidents [12], with mental workload an important parameter for safety, reliability, and efficiency in complex systems [13]. The relationship between workload and fatigue has also been confirmed in pilots. Results from a study of short-haul pilots suggested that higher workloads were associated with slower reaction times and higher levels of fatigue. The fatigue observed in the study may be related to the performance of different short sectors and to the temporal and mental demand of the workload [14].

On the other hand, excessive daytime sleepiness must be differentiated in its diagnosis from fatigue. The causes of daytime sleepiness include insomnia, obstructive sleep apnea, periodic limb movement syndrome, etc. [15]. Its prevalence in the adult population is high and its association with morbidity is significant [16]. Different studies have shown that excessive sleepiness was associated with obesity, cardiometabolic diseases, type 2 diabetes mellitus, and poorer quality of life [17,18,19]. In relation to shift work, there is increasing evidence of its relationship with various sleep disorders such as excessive daytime sleepiness, caused by altered circadian rhythms [20,21], with a high prevalence of daytime sleepiness observed in these type of workers [22]. Furthermore, in the aviation industry where shift work is frequent, a high prevalence of fatigue, sleepiness, and shift work disorder have been observed among airline cabin crew [23].

Given that work overload, fatigue, and daytime sleepiness represent important risk factors in aviation, the objectives of this study were to evaluate the prevalence of these three factors in a sample of Spanish commercial airline pilots and to assess the association between these factors that can negatively affect health and work performance.

## 2. Materials and Methods

### 2.1. Participants

Data were collected from a sample of 283 commercial aviation pilots. Informed consent was obtained from the participants as established by the Helsinki regulations and dictated by the International Medical Association [24]. Data on age and the total flight hours throughout their professional life were collected. Different questionnaires related to work overload, fatigue, and daytime sleepiness were administered. All participants were recruited through the Spanish Association of Commercial Aviation Pilots (COPAC, in its Spanish acronym) and the Spanish Union of Airline pilots (SEPLA, in its Spanish acronym).

### 2.2. Questionnaires

#### 2.2.1. Work Overload Questionnaire (NASA RTLX)

The NASA TLX [25] questionnaire consists of six items that evaluate different dimensions: mental demand, physical demand, temporal demand, performance, effort, and frustration level. These items range from 0 points (low) to 20 points (high), except for the performance item, which scores from 0 points (good) to 20 points (poor). To avoid the weighted procedure, the raw TLX (RTLX) score was calculated. The scores were averaged to calculate an estimate of the overall workload. The use of this score is a common modification of the original scale [26]. 

#### 2.2.2. Fatigue Severity Scale Questionnaire (FSS)

This questionnaire, previously used in other studies in the aviation context [4,27], consists of nine items, with an increasing Likert-type scale ranging from one point to seven points for each one. The total score ranged between 9 and 63 points, although it is necessary to finally calculate the relative score, which is the quotient between the total score divided by the nine items [28]. The relative fatigue score was classified according to Téllez et al. [29]: FSS ≥5 points, there is fatigue; FSS between 4.1 and 4.9 points, doubtful cases of fatigue; FSS ≤4 points, no fatigue. 

#### 2.2.3. Epworth Sleepiness Scale Questionnaire

This questionnaire refers to eight different situations in which a person could fall asleep. These are: “Sitting and reading”, “Watching television”, “Sitting inactive in a public place (e.g., a theater or meeting)”, “As a passenger in a car for an hour without a break”, “Lying down to rest in the afternoon when circumstances permit”, “Sitting and talking to someone”, “Sitting quietly after a lunch without alcohol”, “In a car, while stopped for a few minutes in the traffic”. The punctuation of each item was a Likert-type scale that could vary between 0 and 3 points. The total score ranged between 0 and 24 points and was classified as follows: 0–10 points, normal range in healthy adults; 11–12, mild sleepiness; 13–15, moderate sleepiness; 16–24, severe sleepiness [30].

### 2.3. Statistical Analysis

The Kolmogorov–Smirnov test was used to examine normality. The relationships of the total scores between all questionnaires were studied by the chi-square test. Risk scores (odds ratio) were also obtained between the total scores of work overload, fatigue, and daytime sleepiness. To evaluate the interaction between the different constructs (WO, fatigue, and daytime sleepiness), three multiple linear regression models were applied (enter method) considering the total quantitative score obtained. In each model, one of the construct scores was considered as the independent variable and the other two scores, in addition to age and flight hours, as independent variables. To evaluate the internal consistency of each questionnaire, the Cronbach’s ordinal alpha was calculated. The Cronbach’s alpha was also evaluated to assess whether this parameter improved by removing any of the items of each questionnaire. We also calculated the correlation of each item with the total of the items for each instrument. All the statistical analyses were performed using SPSS statistics v.20 (IBM, Chicago, IL, USA).

## 3. Results

### 3.1. Descriptives

The mean age of the sample was 47.44 ± 9.18 years old. The mean and standard deviation (SD) of flight hours over the course of the professional career of the sample was: 12,363.54 ± 6117.74. Regarding to the WO, 48.0% of the participants were above the 50th percentile and 28.2% above the P75th. For the FSS questionnaire, 21.9% were doubtful cases of fatigue, while 18% presented fatigue. In the case of daytime sleepiness 17.9% were classified as mild sleepiness, 15.8% moderate sleepiness, and 3.9% as severe sleepiness. Table 1 shows the means and SD of the score for each dimension and the total punctuation for the applied questionnaires.

Regarding the work overload questionnaire, the dimensions with the highest scores were mental demand (79.51), temporal demand (78.75), and effort (70.55). In relation to the fatigue questionnaire, the item with the highest average score was item 1 (“My motivation is lower when I am fatigued”), followed by the item “Fatigue interferes with my physical functioning” and “Fatigue interferes with my work, family or social life”. However, the total mean score of the questionnaire was found in the non-fatigue category. Regarding the ESS questionnaire, the total mean score for the whole sample was also in the normal range for the healthy adult category. The items with the highest punctuation were item 5 (“Lying down to rest in the afternoon when circumstances permit”), item 7 (“Sitting quietly after a lunch without alcohol”), and item 2 (“Watching television”).

### 3.2. Association between Fatigue, Work Overload, and Sleepiness 

The association between the different questionnaires was verified. When evaluating this association between the RTLX and ESS questionnaires, an increase in the proportion of pilots with daytime sleepiness was observed when they had a higher WO (when they were above the 50th percentile) (chi-square: 7.32; *p* = 0.005). A total of 45.7% of pilots above the cut-off point for WO had mild, moderate, or severe sleepiness, while those with lower WO and some level of sleepiness were 29.8% [OR: 1.98; CI (95%): 1.21–3.28]. Additionally, the proportion of subjects with fatigue who had higher WO was greater (26.2%) than those who were below the P50 WO (10.2%), with a risk of almost three times higher of suffering from fatigue when the WO increased (chi square:10.99; *p* = 0.001) [OR: 2.97; CI (95%): 1.53–5.77]. Regarding the comparison of proportions between fatigue and daytime sleepiness, significant differences were also found, with a higher proportion of individuals who had mild, moderate, or severe sleepiness having fatigue and the risk being almost twice as high (chi square: 4.74; *p* = 0.023) [OR: 1.96; CI (95%): 1.06–3.62].

Table 2 shows the results of the multiple linear regressions. First, the beta coefficients and the *p*-value of the different variables studied are shown. Significant association between WO with all the independent variables were detected, with all positive except for the total cumulative hours. The total fatigue score was the parameter with the greatest contribution on WO. Likewise, the level of WO was associated with daytime sleepiness and age. When considering daytime sleepiness was the dependent variable of the model, a greater increase in the total score of this questionnaire was observed when fatigue increased. A significant association with WO was also reported. Multiple regression analysis on fatigue showed that WO had the strongest effect on fatigue, followed by sleepiness and flight hours. Predictors of fatigue were, in order of importance, work overload, total flight hours, and sleepiness.

### 3.3. Internal Consistency

The FSS (Cronbach’s alpha: 0.873) and the ESS (Cronbach’s alpha: 0.729) questionnaires showed good alpha measures of internal consistency. When the Cronbach’s alpha was evaluated by eliminating each of the items of both questionnaires, there was no significant improvement in the alpha value. For the RTLX questionnaire, it was observed that by eliminating the performance item, the Cronbach’s alpha increased substantially, becoming acceptable. This is because in the case of this sample of pilots, performance was almost always excellent because they understood that it was based on the ability to complete the task (i.e., landing the airplane without any mishap). For this reason, the performance item was eliminated and, therefore, the Cronbach’s alpha for the questionnaire was 0.665 (Table 3). 

## 4. Discussion

In this paper, we examined the WO, fatigue, and daytime sleepiness among Spanish commercial aviation pilots through different instruments validated by the literature [22,31,32,33,34]. WO is a factor that has been studied among different professional groups as in health care, transportation, or aviation [35,36,37,38]. In this context, there are studies that have evaluated the perceptions of a pseudo-pilot’s workload used in simulators to train air traffic controllers [39]. Additionally, Alaimo et al. [40] compared subjective and objective measurements of the pilot’s workload in different flight phases whereas Morris and Leung et al. [41] investigated the effects of increasing the mental demands on aircrew performance. Similarly, different tasks were evaluated under different mental workload levels by flight simulation by considering subjective measures such as NASA-TLX and other physiological parameters such as the use of electrocardiographs [42]. Additionally, Dahlstrom and Nahlinder [43] observed that in certain maneuvers such as takeoff, rejected take-off, engine failure, or landing, the mental demand is remarkably high.

In line with other authors, in this study, we evaluated the total score and its different dimensions of the NASA-TLX questionnaire [26,44], so we could figure out which items contributed more significantly to overload in the job environment. In the present study, the highest scores were obtained for mental and temporal demand and effort. Comparing our results with those obtained in another sample of Spanish professionals from different fields [45], mental and temporal demand and effort remained higher. However, when we look at specific professional groups, teachers or health care personnel have similar or even higher scores of the items analyzed [45]. In another study conducted in intensive care unit nurses at seven hospitals, similar levels were found for mental demand (nurses: 76.15 ± 21.59 vs. pilots: 79.51 ± 15.67) and effort (nurses: 71.27 ± 20.73 vs. pilots: 70.55 ± 16.75), however, the score for frustration level was higher in the health care workers (nurses: 43.77 ± 26.09 vs. pilots: 36.35 ± 23.27), while the temporal demand was lower (nurses: 62.98 ± 26.04 vs. pilots: 78.75 ± 18.96) [31]. The item with the lowest score was performance, however, this dimension disturbed the results since the Cronbach’s alpha was acceptable when this item was eliminated. A possible explanation for this could be that in this sample of commercial aviation pilots considered that their performance was almost always very good since the most important priority is that the aircraft reaches its destination without any mishap.

As shown in a recent meta-analysis, burnout, a term that is equivalent to exhaustion resulting from a high work overload, is associated with cognitive impairment related to executive function, working memory, attention, and processing speed, among others. It increases as overload does, and when jobs demand greater attentional and executive control [46]. Similarly, it has been proven that medium-high mental workload environments can disrupt the pilots’ ability to hear, understand, and respond to auditory messages [41]. However, not only do cognitive impairments occur because of continued exposure to burnout, but it is also associated with the risk of comorbidities such as systemic inflammation, immunosuppression, metabolic syndrome, cardiovascular diseases, and premature death [47,48]. It also affects the quality of life and mental health [49].

It has been established that burnout and sleep disorders could have bidirectional effects, for example, in medical students, emotional exhaustion and daytime sleepiness showed a significant mutual influence on each other [50]. Additionally, Armon et al. [51] reported that burnout predicted future insomnia and insomnia predicted future burnout in a prospective study of employed adults. To support this relationship, sleepiness and fatigue were measured in a group experiencing severe occupational burnout. The outcome was sleeping fragmentation, more wake time, or lower sleep efficiency, and increased sleepiness and daytime fatigue were found, suggesting that job burnout is characterized by sleep impairment [52]. In addition, in a prospective longitudinal study involving 380 medicine residents in a clinic of Minnesota, an association was observed between self-reported errors and the EES score. The ESS mean score of those who reported errors was 9.58 [CI:95% 0.66 (−0.62 to 1.94)], very similar to the mean observed in the present sample (9.51 ± 3.65). Additionally, the authors suggested that fatigue, sleepiness, and exhaustion between others were independently associated with the risk of future self-perceived medical errors [53]. In addition, in a cross-sectional study involving 251 seafarers who were exposed to different stress factors, they found that a single question on the subjective perception of sleep duration may be more appropriate to evaluate the burnout risk than the ESS score [54]. In contrast, in the present study, we observed that the level of work overload was affected by daytime sleepiness.

In relation to fatigue, some of the triggering factors include loss of sleep, extended wakefulness, circadian phase irregularities and workload [55]. For example, in a study analyzing the perception of fatigue in short- and long-haul flight pilots, the main cause of fatigue was related to sleep loss. Among the short-haul pilots, multi-leg flights and early mornings contributed to increased fatigue, in addition to the number of legs per day, time constraints, and consecutive workdays. In contrast, night flights and jet lag were the main causes of fatigue for long-haul pilots [56]. Regarding the effects of fatigue, one of them is impaired alertness. When fatigue is present, individuals may begin to feel drowsy, and when this is excessive, it can lead to short periods of uncontrollable sleep (microsleeps). Additionally, fatigue can lead to a decrease in performance and may also have long-term effects on health [55]. Additionally, sleep deprivation negatively impacts self-reported mood in subjective and objective fatigue and, regarding their competencies, and cognitive flexibility and hand-eye coordination declines when the time awake increases [57]. Likewise, it has been observed that fatigue risks increase sleep problems and stress [27].

This study was not without limitations. This was an observational study and therefore, it was not possible to establish causality between the variables studied. On the other hand, it was a relatively small sample, which may not be representative of all Spanish aviation pilots. Likewise, participation in the study was voluntary, so it is possible that there exists bias toward those individuals who were more concerned about their health, although the importance of taking part in the research was explained to the group, regardless of health conditions. In addition, the Cronbach’s alpha for the RTLX questionnaire was questionable as it was slightly below the 0.7 cut-off point that is considered acceptable. Given the exploratory nature of the study, the results derived from this questionnaire are shown, although they should be interpreted with caution. It is also likely that the context in which the evaluation is carried out is affects the reliability. The test may not have the same accuracy when assessing workload under a common and concrete scenario (e.g., simulator activities) as when the subjects are in different conditions (different types of flight, type of aircraft, etc.). Similarly, reliability may be compromised if the test is applied immediately after a work session or if it is applied retrospectively, as in this case, the participants may have forgotten important aspects of the workload. Finally, no information was collected on other mood-related factors that could affect the variables analyzed.

On the other hand, the study had strong points such as the recollection of numerous variables in a group that is difficult to access and not very accustomed to supplying data related to physio psychological parameters such as WO, fatigue, and sleepiness. Additionally, these are parameters that have been little studied among Spanish aviation pilots. These results can provide relevant information to improve the pilot conditions and safety.

## 5. Conclusions

This study investigated the prevalence and its association of three important factors in aviation. The three elements that contribute most to WO are, first, the mental demand, second, the temporal demand or pace imposed to perform the tasks, and finally, the effort made to achieve optimal performance. Although the mean scores obtained in the fatigue and sleepiness questionnaires were within the healthy categories, almost 20% of the pilots presented fatigue, and in the same proportion moderate or severe sleepiness. We observed that when the WO was above the 50th percentile, the risk of sleepiness doubled and the risk of fatigue tripled. We have shown that WO, fatigue, and daytime sleepiness are closely associated. Along this line, we noted that the total fatigue was the most influential parameter on WO and at the same time, WO had the strongest effect on fatigue, followed by sleepiness. For this reason, these three factors related to pilot health and aviation safety could be evaluated together. We believe that it is important to continue with this line of work and to conduct broader longitudinal studies that will allow us to delve into the underlying causes of workload, fatigue, and sleepiness in aviation.

## Figures and Tables

**Table 1:** Punctuation of each item and the total score for the RTLX, FSS, and ESS questionnaires for the total sample.

Questionnaire	Items	Score (Mean ± SD)
Work Overload (RTLX) (1) (N = 271)	Mental demand	79.51 ± 15.67
	Physical demand	45.00 ± 21.44
	Temporal demand	78.75 ± 18.96
	Performance	17.52 ± 13.38
	Effort	70.55 ± 16.75
	Frustration	36.35 ± 23.27
	Total score	54.62 ± 10.51
Fatigue Severity Scale (FSS) (2) (N = 283)	My motivation is lower when I am fatigued	5.12 ± 1.60
	Exercise brings on my fatigue	2.82 ± 1.57
	I am easily fatigued	2.28 ± 1.32
	Fatigue interferes with my physical functioning	4.75 ± 1.61
	Fatigue causes frequent problems for me	2.81 ± 1.63
	My fatigue prevents sustained physical functioning	3.45 ± 1.89
	Fatigue interferes with carrying out certain duties and responsibilities	4.17 ± 1.89
	Fatigue is among my three most disabling symptoms	4.08 ± 2.10
	Fatigue interferes with my work, family, or social life	4.27 ± 1.90
	Total score	3.75 ± 1.22
The Epworth Sleepiness Scale (ESS) (3) (N = 279)	Sitting and reading	1.48 ± 0.857
	Watching television	1,51 ± 0.823
	Sitting inactive in a public place (e.g., a theater or meeting)	0.75 ± 0.638
	As a passenger in a car for an hour without a break	1.08 ± 0.925
	Lying down to rest in the afternoon when circumstances permit	2.23 ± 0.828
	Sitting and talking to someone	0.39 ± 0.511
	Sitting quietly after a lunch without alcohol	1.64 ± 0.937
	In a car, while stopped for a few minutes in the traffic	0.42 ± 0.556
	Total score	9.51 ± 3.65

(1) Work Overload RTLX questionnaire [26]; (2) Fatigue Severity Scale questionnaire (FSS) [27]; (3) Epworth Sleepiness Scale Questionnaire (ESS) [29].

**Table 2:** Association model between fatigue, work overload, and sleepiness.

	Work Overload		Sleepiness		Fatigue	
Independent Variable	βeta	*p*-Value	βeta	*p*-Value	βeta	*p*-Value
Age	0.249	0.007	−0.22	NS	−0.125	NS
Total flight hours	−0.230	0.013	0.012	NS	0.182	0.05
Work overload (1)	-	-	0.129	0.042	0.271	0.000
Fatigue (2)	0.271	0.000	0.176	0.005	-	-
Sleepiness (3)	0.120	0.042	-	-	0.164	0.005

(1) Work Overload RTLX questionnaire [26] (2) Fatigue Severity Scale questionnaire (FSS) [28]; (3) Epworth Sleepiness Scale Questionnaire (ESS) [30]. NS: non-significant.

**Table 3:** Reliability analysis. Cronbach’s alpha if each item is deleted and the item-total correlation across the items of the RTLX, Fatigue Severity Scale, and Epworth Sleepiness Scale questionnaires.

Questionnaire	Items	Cronbach Alpha If Item Is Deleted	Corrected Item-Total Correlation
Work overload RTLX (1)	Mental demand	0.460	0.501
	Physical demand	0.551	0.277
	Temporal demand	0.488	0.403
	Performance	0.665	−0.142
	Effort	0.445	0.5.19
	Frustration	0.512	0.357
	Total Cronbach’s alpha	0.575	
	Total Cronbach’s alpha when performance is eliminated	0.665	
Fatigue Severity Scale (FSS) (2)	My motivation is lower when I am fatigued	0.866	0.521
	Exercise brings on my fatigue	0.887	0.245
	I am easily fatigued	0.871	0.452
	Fatigue interferes with my physical functioning	0.858	0.622
	Fatigue causes frequent problems for me	0.849	0.725
	My fatigue prevents sustained physical functioning	0.852	0.681
	Fatigue interferes with carrying out certain duties and responsibilities	0.848	0.723
	Fatigue is among my three most disabling symptoms	0.845	0.750
	Fatigue interferes with my work, family, or social life	0.844	0.754
	Total Cronbach’s alpha	0.872	
The Epworth Sleepiness Scale (ESS) (3)	Sitting and reading	0.682	0.509
	Watching television	0.706	0.400
	Sitting inactive in a public place (e.g., a theater or meeting)	0.698	0.452
	As a passenger in a car for an hour without a break	0.695	0.459
	Lying down to rest in the afternoon when circumstances permit	0.709	0.389
	Sitting and talking to someone.	0.709	0.410
	Sitting quietly after a lunch without alcohol	0.699	0.445
	In a car, while stopped for a few minutes in the traffic	0.713	0.370
	Total Cronbach’s alpha	0.729	

(1) Work Overload RTLX questionnaire [26] (2) Fatigue Severity Scale questionnaire (FSS) [28]; (3) Epworth Sleepiness Scale Questionnaire (ESS) [30].

## Data Availability

The dataset used during the current study is available from the corresponding author upon reasonable request.
